# Molecular Analysis of Tigecycline Resistance in Carbapenem-Resistant Enterobacterales (CRE) in Mthatha and Surrounding Hospitals

**DOI:** 10.3390/antibiotics14040407

**Published:** 2025-04-16

**Authors:** Luyolo Vumba, Ravesh Singh, Sandeep Vasaikar

**Affiliations:** 1 Department of Laboratory Medicine and Pathology, Division of Medical Microbiology, Faculty of Medicine & Health Sciences, Walter Sisulu University, Mthatha 5117, South Africa; luyolovumba24@gmail.com; 2 Department of Medical Microbiology, National Laboratory Service, Inkosi Albert Luthuli Hospital, Durban 4001, South Africa; singhra@ukzn.ac.za

**Keywords:** tigecycline resistance, E-test, CRE infections, PCR, tigecycline resistance risk factors, *tet(X)* genes, carbapenemase genes

## Abstract

**Background:** The emergence of carbapenem-resistant *Enterobacterales* is prevalent and poses a significant threat to health systems worldwide. This study aimed to conduct a molecular analysis of tigecycline resistance in 100 CRE isolates from Mthatha Hospital and surrounding hospitals. **Methods**: A retrospective study among patients who attended Nelson Mandela Academic Hospital (NMAH) and Mthatha Regional Hospital (MRH), Eastern Cape, South Africa. *Enterobacterales* isolates were identified using the Vitek2^®^ system (bioMérieux); an E-test was performed on 100 CRE isolates according to the manufacturer’s instructions. PCR assays for rapid detection of *tet(X)* and its variants, including *tet(X1)* and *tet(X2)*, and high-level tigecycline resistance genes *tet(X3)*, *tet(X4)*, and *tet(X5)* were developed. **Results:** The results show a notably high prevalence of CRE infections in neonatal, male surgical, and maternal and pediatric wards, predominantly driven by *Klebsiella* species (53.4%), followed by *Enterobacter* species (20.5%) and then *Escherichia coli* (6.7%), and 7.2% of CRE isolates were resistant to tigecycline (E-test). In this study, *tet(X)* genes were not identified as the primary mechanism of tigecycline resistance. The risk factors associated with tigecycline resistance in CRE include age, pre-exposure to antibiotics, prolonged hospitalization, and undergoing invasive procedures, indicated by strong r = 0.9501. **Conclusions:** CRE gradually evolves, posing a significant threat to patients of all ages; early detection of carbapenemase production in clinical infections, carriage states, or both is essential to prevent hospital-based outbreaks.

## 1. Introduction

The emergence of carbapenem-resistant *Enterobacterales* is prevalent and poses a significant threat to health systems worldwide [1]. There is an increment of about 50–60% in the mortality rate among patients with CRE bloodstream infections reported by the European Centers for Disease Prevention and Control [2]. The most common CRE that are responsible for a wide range of infections (urinary tract, pneumonia, soft tissue bloodstream infection, wound infection, meningitis, septicaemia, endocarditis, severe intra-abdominal infections, and lower respiratory tract infections) in clinical settings are *Escherichia coli*, *Klebsiella pneumoniae*, *Salmonella* spp., *Serratia marcescens*, *Enterobacter* spp., and other Gram-negative bacteria [3]. The first CRE case was reported in 2011 in Gauteng province with *Klebsiella pneumoniae carbapenem (KPC)* and *New Delhi metallolactamase-β-1 (NDM-1)* as carbapenem-resistant genes, followed by many others in 2012 [4]. A study conducted by Hovan et al. (2021) reported a mortality rate of about 32% in patients with CRE bloodstream infections within 14 days, even higher in critically ill patients, cancer patients, and immunocompromised patients [5]. Prolonged unnecessary stays at hospitals, higher mortality rates, and higher morbidity rates have increased worldwide and are caused by nosocomial carbapenem-resistant *Enterobacterales* (CRE) outbreaks [2]. Carbapenems were a last resort to treat CRE infections, but due to misuse, overuse, and other resistance mechanisms acquired by CRE, the drug of choice had to be changed. There are two ways in which CRE acquires resistant mechanisms (intrinsic and acquired), and both play a significant role in the drastic rise in CRE resistance. For the intrinsic mechanism, there is a development of carbapenemases from class A (serine carbapenemases), while in the acquired mechanism, the main development is of efflux pumps, and efflux pumps are mostly reported in *Enterobacter cloacae*, *Serratia marcescens*, and *Klebsiella* spp. [6]. Most of the acquired mechanisms are mediated by plasmids and associated with horizontal gene transfer. Common carbapenemases are linked to acquired mechanisms, with New Delhi metallo-lactamase (NDM) now reported as the most prevalent in clinical isolates [7]. *KPC* is one of the most common genes in clinical isolates and has caused multiple nosocomial outbreaks worldwide [8]. About 50.4% of organisms causing infections in South Africa are CRE. South Africa has a relatively high number of CRE infections compared to other African countries but has fewer CRE infections compared to European countries (70–80%). CRE have developed resistant strains to almost all classes of antibiotics available, which makes it difficult for clinicians to select an appropriate and effective drug to treat and reduce infections and outbreaks caused by CRE [9]. Fosfomycin, clindamycin, and cotrimoxazole were successfully used to treat CRE infections, but after some time, limitations such as nephrotoxicity in patients with renal complications were reported, and new, effective/appropriate drug options to treat CRE infections had to be developed [10]. In 2010, colistin and tigecycline became last-resort treatment options for CRE and were made available in clinical practice depending on the country and its antibiotic guidelines; for instance, in South Africa, both antibiotics are reserved for specific clinical cases [11]. Tigecycline is one of the antibiotics developed to treat MDR infections, including infections caused by CRE, and it belongs to the tetracycline class [12,13]. For over a decade, tetracycline has been used to successfully treat infections in both animals and humans. However, only 25% of tetracycline is absorbable after intake and 75% is excreted as metabolites, according to [14], and tigecycline is administered only by intravenous infusion over 30 to 60 min. Over time, pathogens developed resistant mechanisms against the tetracycline class, resulting in the tetracycline class being not completely effective. Scientists structurally modified tetracycline and produced tigecycline, a drug with unique anti-bacterial properties that enable it to treat multi-drug-resistant infections successfully. The first member of the glycylcycline class of antibiotics to display vital properties in vitro against multi-drug resistant bacteria, including CRE, is tigecycline. Some of these properties include its ability to overcome typical resistance mechanisms that confer bacteria resistance to the tetracycline class; additionally, it has a different binding orientation that allows it to bind with high affinity to bacterial ribosomes, and it prevents protein synthesis of bacteria by interfering with aminoacyl-tRNA, which usually binds with ribosomes and allows bacteria to multiply. Eventually, bacterial growth is inhibited by binding to the bacterial 30S ribosome [15,16,17]. Another name for tigecycline is GAR-936 (third generation of tetracycline class of antibiotics) because it was derived from the addition of 9-tert-butyl-glycylamido to minocycline. Tigecycline overcomes the standard resistant mechanisms of CRE such as efflux pumps and ribosomal protection mechanisms [18]. The Food and Drug Administration approved tigecycline for treating patients with complicated skin and skin structure infections (cSSTI), complicated intra-abdominal infections (cIAI), and community-acquired bacterial pneumonia, but recommended it not be used in patients with diabetic foot infection in 2005 [17]. All infections caused by *Clostridium difficile* are successfully treated using tigecycline. Due to the broad spectrum of most pathogens that are resistant to first-line antibiotics, clinicians now use it off-label to treat ventilator-associated pneumonia (VAP), hospital-acquired pneumonia (HAP), and bloodstream infections (BSI) caused by pathogens, particularly carbapenem-resistant (CR) bacteria [16]. Pathogens have developed resistance mechanisms against tigecycline, even though it is a last resort for treating infections caused by MRD and XDR, and tigecycline resistance has been reported globally [13]. Tigecycline resistance in CRE is associated with increased mortality and morbidity [17]. Antimicrobial sales are predicted to increase by 11.5% from 2017 to 104,079 tons globally in 2030 [18]. Tigecycline resistance is not only a healthcare system burden but also an economic burden that needs the world’s immediate intervention because tigecycline resistance would need more healthcare personnel to be employed, adequate infrastructure to accommodate patients, and preventative measures to control tigecycline outbreaks [19,20]. This study aimed to conduct a molecular analysis of tigecycline resistance in 100 CRE isolates from Mthatha Hospital and surrounding hospitals. The study is based on the null hypothesis that there is no tigecycline resistance in CRE isolates collected at Mthatha Hospital.

## 2. Results

### 2.1. Carbapenem Resistant Enterobacterales Species

**Figure 1:**
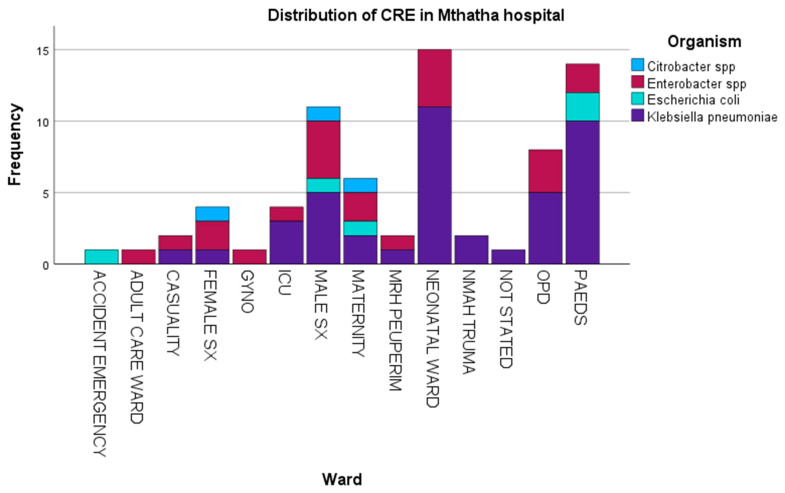
The bar chart represents the distribution of CRE (carbapenem-resistant Enterobacterales) infections in different wards of Mthatha Hospital, with various organisms identified using different colors.

**Figure 2:**
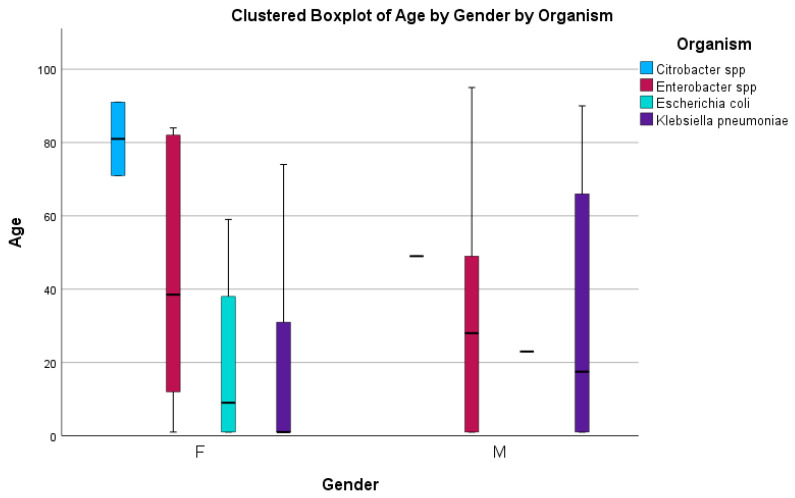
A boxplot showing CRE distribution by gender.

The first objective was to determine carbapenem-resistant Enterobacterales’ prevalence in Mthatha Hospital and surrounding hospitals. The CRE prevalence graph charts were obtained using SPSS version 2.0 (Figure 1). They were produced from a descriptive analysis (frequency) because a categorical variable (organism) against frequency was investigated. A boxplot (Figure 2) was made because a relationship between two categorical variables (sex and CRE organism) and one continuous variable (age) was explored. The bar chart in Figure 1 represents the distribution of CRE (carbapenem-resistant Enterobacterales) infections in different wards of Mthatha Hospital, with various organisms identified using different colors. The pathogens identified include *Klebsiella pneumoniae*, *Escherichia coli*, *Enterobacter* spp., and *Citrobacter* spp.

Table 1 shows that there is a notably high prevalence of CRE infections in the neonatal, male surgical, and maternity and pediatric wards, predominantly driven by *Klebsiella* spp. (53.4%), followed by *Enterobacter* spp. (20.5%), *Citrobacter* spp. (19.4%), and *Escherichia coli* (6.7%). *Klebsiella* spp. is the most common organism across multiple wards, indicating its dominance in causing infections. *Enterobacter* spp. and *Escherichia coli* were present in several wards but at significantly lower frequencies. The accident and emergency department, adult care ward, and outpatient department exhibit relatively low occurrences of CRE infections.

The boxplot chart shows age distribution by gender, categorized by different CRE organisms in a clustered format.

No gender and age differences in CRE distribution were observed for females; most organisms have a relatively compact age distribution, except for a few outliers. *Klebsiella pneumoniae* was reported more in the comprehensive age range for both genders, with a few exceptions in younger individuals. *Citrobacter* spp. is present primarily in older age groups, particularly for females; *Enterobacter* spp. has a relatively younger distribution for males and females, with a narrow range. Most *Escherichia coli* cases were observed in young females. There were exceptional cases observed in both genders, indicating that some patients fall outside the usual age ranges affected by specific pathogens.

A RESIST-4 OKNV test was used to detect the positive carbapenemase gene found on Mthatha CRE isolates. Table 2 summarizes the prevalent carbapenemase genes accordingly. There were no KPC and VIM genes detected (0%) on our CRE; the most prevalent carbapenemase was NDM (54.2%), mostly detected on *Klebsiella* spp. and *E. coli*, followed by OXA-48 (30.6%), mostly detected on *E. coli* and *Klebsiella* spp. Also, about 15.2% of CRE isolates were negative (no gene detected).

### 2.2. Tigecycline Susceptibility Using E-Test

Tigecycline susceptibility was determined using an E-test (Etest, bioMérieux, France). The bar graph chart was produced using SPSS version 20 to represent the results obtained. Since a relationship between one dependent categorical variable (tigecycline susceptibility) and one continuous independent variable (age) was explored, a descriptive analysis (frequency) was an appropriate tool to use. The chart shows tigecycline susceptibility in different age groups, categorized into three susceptibility levels: S (susceptible), R (resistant), and I (intermediate). The data are represented as bars, with error bars indicating each measurement’s 95% confidence interval. Table 3 is a chi-square table showing tigecycline resistance by age group in Mthatha Hospital and surrounding hospitals.

**Figure 3:**
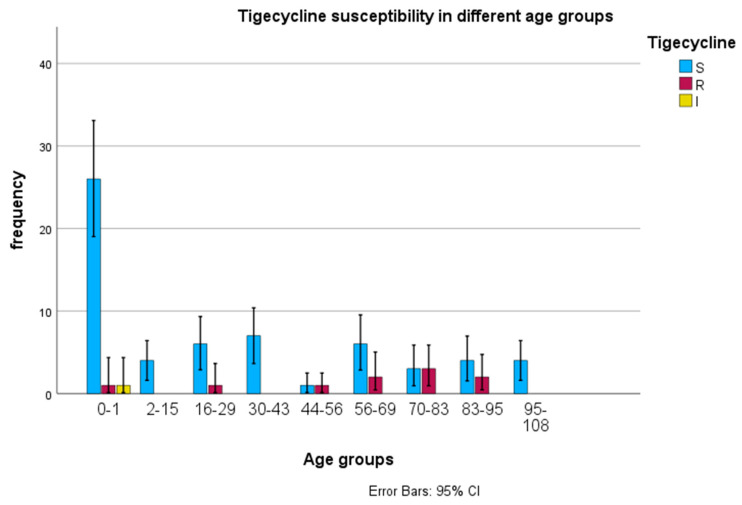
Tigecycline susceptibility in different age groups of Mthatha and surrounding hospitals. S, susceptible; R, resistant; I, intermediate resistant.

As can be observed from the graph, tigecycline resistance increases with age, except in a few cases with no resistance. The E-test showed a tigecycline susceptibility of 92.8%, a resistance of 5.8%, and 1.5% intermediate resistance. The 0–1 age group shows a high level of tigecycline susceptibility (S) compared to other age groups, and relatively low tigecycline resistance (R) and intermediate resistance (I). In age groups 2–15, 16–29, 30–43, and onwards, the frequency of susceptibility decreases notably compared to the 0–1 age group.The frequency of resistance (R) and intermediate (I) levels become more noticeable, although still lower than susceptible (S) levels. In older age groups (44–56 and beyond), there is a trend of lower tigecycline susceptibility rates with some variability across different age groups. Resistance (R) and intermediate (I) levels appear relatively consistent but remain lower than susceptible (S) levels.Statistical analysis was conducted to explore the association between tigecycline resistance (categorial variable) and age (categorical variable, age was grouped) to evaluate the statistical significance. Table 4 is a Chi-square table showing tigecycline resistance by age group at Mthatha and surrounding hospitals. Tigecycline resistance is present in both children (neonates) and adult patients, slightly more in adults than children. Figure 3 summmarises tigecycline susceptibility in different age groups of Mthatha and surrounding hospitals. S, susceptible; R, resistant; I, intermediate resistant.

The difference in tigecycline resistance between age groups is statistically insignificant, as indicated by a *p*-value of 0.944. Based on this sample, there is no evidence that tigecycline resistance is associated with age, even though it is mostly seen in adult patients.

### 2.3. Detection of tet(X) Genes Using PCR

**Figure 4:**
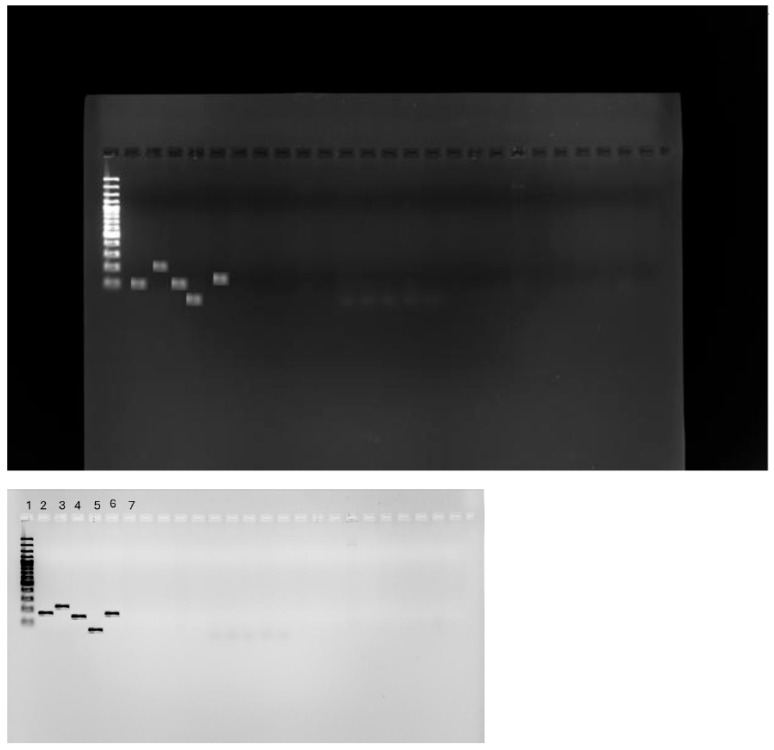
Agarose gel electrophoresis picture of tigecycline *tet(X)* genes, DNA ladder on lane 1, with a positive control for each gene, *tet(X1)* with 141 base pairs (lane 2), *tet(X2)* with 227 base pairs (lane 3), *tet(X3)* with 125 base pairs (lane 4), *tet(X4)* with 93 base pairs (lane 5), and *tet(X5)* with 161 base pairs (lane 6).

A multiple SYBR green-based PCR assay was developed for the detection of five *tet(X)* variant genes from different samples, namely *tet(X1)*, *tet(X2)*, *tet(X3)*, *tet(X4)*, *and tet(X5)*, which were also used as positive controls for each gene. Primer Premier 5.0 (PRIMIER Biosoft International, Palo Alto, CA, USA) was used to design specific primers for *tet(X1)*, *tet(X2)*, *tet(X3)*, and *tet(X4)*, as well as a universal *tet(X5)* primer set. Of the 100 CRE clinical isolates studied, no *tet (X)* gene was detected that was responsible for tigecycline resistance. This was expected since both phenotypic methods used showed lower tigecycline resistance, <15%. The DNA extracts were nanodropped and the concentration of each extract was determined, and isolates with <1.81 concentration were qualified until the required DNA concentration was met. Figure 4 shows the PCR result viewed with a UV light machine.

### 2.4. Risk Factors Associated with Tigecycline Resistance

To determine the risk factors associated with tigecycline resistance in CRE at Mthatha Hospital and surrounding hospitals, multiple linear regression was used to determine *p*-values and R-values, as shown in Table 5 below. The R-value could not be determined for other risk factors, such as pre-exposure to antibiotics, invasive procedures, and hospitalization duration. The correlation between tigecycline resistance and wards was weak, indicated by r = 0.4182, and the *p*-value of 0.1631 suggested that the association was not statistically significant; for age, a statistically significant *p*-value of 0.001 suggests a strong relationship between tigecycline resistance and age group, and the r = value of 0.9501 indicates a very strong correlation. There was a positive correlation between tigecycline resistance and pre-exposure to antibiotics, as indicated by a statistically significant *p*-value of 0.0478. Undergoing specific invasive procedures might contribute to tigecycline resistance, as indicated by a statistically significant *p*-value of 0.0478. A non-significant *p*-value of 0.3679 was found between the length of hospitalization and tigecycline resistance.

## 3. Discussion

### 3.1. Determining the CRE Species

Carbapenem-resistant *Enterobacterales* (CRE) is a global public health concern affecting communities and healthcare facilities [21,22]. The worldwide increase in CRE infections is alarming, given their association with high mortality rates and their substantial economic burden in a clinical setting [23,24,25]. Carbapenem resistance among *Enterobacterales* in South Africa has historically been relatively low and stable. The overuse and misuse of these antibiotics can lead to resistance, making infections more challenging to treat and posing a significant threat to public health [26]. This study explored the prevalence of CRE infections in Mthatha Hospital and surrounding hospitals. The study involved a comprehensive review of patient records and laboratory data to identify cases of CRE infection. The prevalence was then represented in a graph chart using SPSS. The results show a notably high prevalence of CRE infections in the neonatal, male surgical, and maternal and pediatric wards, predominantly driven by *Klebsiella* spp. (53.4%), followed by *Enterobacter* spp. (20.5%) and then *Escherichia coli* (6.7%). These findings are in agreement with findings obtained in previous studies conducted in community hospitals in Bahrain (87%), Taiwan, and Nigeria (91%) [26,27,28]. This is because *K. pneumoniae* has been reported worldwide as a dominant species; hence, a carbapenem-resistant *K. pneumoniae* (CRKP) gene is reported. The dominance of *Klebsiella* spp. in causing infections is significant as it indicates the need for targeted interventions to control the spread of CRE. This shows that neonatal wards are significantly affected by CRE infections because neonates have weak immune systems to fight off CRE infections. Male surgical and maternity wards have a broad diversity of CRE infections; hence, multiple organisms were detected. This might be because of the high number of immunocompromised patients in these wards. Usually, these wards have a population that has been pre-exposed to tetracycline antibiotics and has undergone different surgeries, which are risk factors for acquiring CRE infection [29]. *Klebsiella* spp. is the most common organism across multiple wards, indicating its dominance in causing infections; *Enterobacter* spp. and *Escherichia coli* are present in several wards but at significantly lower frequencies. The accident and emergency department, adult care ward, and outpatient department exhibit relatively low occurrences of CRE infections. These findings are significant in South Africa’s healthcare system as they highlight the urgent need for targeted interventions and increased surveillance to control the spread of CRE infections. In this study, the age distribution was skewed toward older patients when CRE infections were analyzed; *Klebsiella* spp. was prevalent in a comprehensive age range for both genders, with some exceptional cases in younger individuals; *Citrobacter* spp. is primarily present in older age groups, particularly for females; *Enterobacter* spp. has a relatively younger age distribution for both males and females, with a narrow range. As age increases, the variety of CRE organisms also increases; this might be because older patients are affected by more than one CRE acquisition risk factor, leading to the high prevalence of CRE in wards occupied by older people [19]. The outcome of this study shows that the prevalence of CRE infection in Mthatha Hospital and surrounding hospitals is 30%. This estimate is lower than the estimates reported in the literature of other countries, such as the United States (32%) and India (38%) [30], but higher than estimates that were reported in Tunisia (15.8%) and other countries such as China and Nigeria, which reported varying numbers of CRE infection prevalence [31]. This could be because the population sizes of the studies were different, and the study sites were in other regions of the world with different economic statuses and clinical settings. Three central resistant genes of carbapenems have been reported in clinical isolates worldwide, namely *KPC*, *NDM*, and *OXA-48*, with *KPC* being the most prevalent [32]. This study’s most identified class A genes are *NDM* (56.9%) and *OXA-48* (31.9%); 11.1% were negative. These findings are consistent with the outcome reported by Park et al. (2020) [24], who investigated the prevalence of CRE in clinical isolates, and NDM (5.1%) was reported, followed by *Oxa-48* (26.8%). The outcomes of this study differ from those of Lee et al. [33], who evaluated CRE prevalent genes in clinical isolates, and *VIM* (80%) was the most prevalent gene. This may be due to the different years of reporting; sometimes, specific genes are prevalent in a particular year because of reported outbreaks [34].

### 3.2. Tigecycline Susceptibility Using E-Test

The emergence of multi-drug-resistant bacteria in hospitals significantly limits the therapeutic options to treat life-threatening infections and forces clinicians to use last-resort antibiotics, including tigecycline [35]. Since CRE infections are prevalent and are associated with increased mortality and morbidity worldwide, the WHO, the FDA, and EUCAST recommend clinicians use colistin and tigecycline to treat these infections [36]. This study focuses more on tigecycline and its effectiveness against CRE. Tigecycline is the first member of the glycylcycline class of antibiotics that has displayed important properties in vitro against CRE and is a last resort among treatment options for CRE; hence, it is crucial to study its antibiotic susceptibility [15,17,37]. Several methods are used to perform tigecycline antibiotic susceptibility tests (AST), but the most reliable and accurate ones include disk diffusion and the E-test [38]. Many factors affect tigecycline susceptibility, such as the unavailability of predictive breakpoints to interpret results for some pathogens on CLSI. The present study performed tigecycline AST using an E-test on 100 CRE isolates in South Africa from Mthatha Hospital and surrounding hospitals. We found that 92.8% of CRE isolates were susceptible to tigecycline, 1.5% had intermediate resistance to tigecycline, and 5.8% were resistant to tigecycline. Tigecycline resistance is present in children (neonates) and adult patients, slightly more in adults than children. The difference in tigecycline resistance between age groups is statistically insignificant, as indicated by a *p*-value of 0.944. Based on this sample, there is no evidence that tigecycline resistance is associated with age, even though it is mainly seen in adult patients. The age group 0–1 shows a high level of tigecycline susceptibility (S) compared to other age groups; there is relatively low tigecycline resistance (R) and intermediate resistance (I). In age groups 2–15, 16–29, 30–43, and onwards, susceptibility frequency decreases notably compared to the 0–1 age group. As age increases, tigecycline resistance also increases; this is justified by the fact that as people age, they are likely to go to healthcare facilities, use antibiotics (especially tetracyclines), undergo surgeries, encounter others who have been admitted to ICUs, and have underlying health conditions, which are prime risk factors for tigecycline resistance [26,29,39]. These outcomes were consistent with outcomes reported by Wilson et al. (2023) [40], where 75.3% tigecycline susceptibility was obtained on CRE hospital isolates using the E-test method; the study was conducted in India. Li et al. [17] conducted a similar study in China, where tigecycline AST was performed on CRE, and 82% tigecycline susceptibility was reported using KB diffusion. Yu et al. [41] conducted an AST on CRE and reported 80–90% tigecycline sensitivity in critically ill patients. Lower tigecycline resistance in other countries may be because tigecycline is a reserved drug; the WHO recommends the use of this drug with certain specifications to prevent the overuse and misuse of tigecycline that could lead to increased resistance. The outcomes of this study are not aligned with the study conducted by Heidary et al. [42], where tigecycline AST was performed using the disk diffusion method on 51 CRE hospital isolates, and 30 (58%) were resistant to tigecycline. This significant difference might be because of the difference in sample size and different screening methods employed to perform AST. Tigecycline is administered only by intravenous infusion over 30 to 60 min. The recommended tigecycline dose is 100 mg on the first day, followed by 50 mg every 12 h on subsequent days. The commonly reported adverse effects from tigecycline use include nausea and vomiting [40]. According to Viechtbauer et al. [43], tigecycline is equivalent to imipenem in abdominal and intra-abdominal infections and to a combination of aztreonam and vancomycin in skin and skin structures. Tigecycline remains a promising and effective drug for CRE treatment. The reasons for substituting TG with equivalent drugs include the following: (1) Tigecycline possesses an extensive range of action against bacteria, including oxygen-requiring and oxygen-averse types and Gram-negative and Gram-positive varieties. (2) It is potent against severe infections like those resistant to vancomycin among *Enterococcus* species (VRE), Methicillin-resistant *Staphylococcus aureus* (MRSA), antibiotic-resistant *Streptococcus pneumoniae*, and Gram-negative respiratory pathogens. (3) It is effective in treating a variety of diseases caused by pathogens. (4) Tigecycline shows efficacy against less common bacterial infections, such as those caused by Mycoplasma pneumonia. (5) It is safe for use in patients with renal and hepatic insufficiency and the elderly without the need to adjust the dose [44]. Other studies reported ribosome protection, drug-degrading enzymes, cell membrane pore channel protein modification, and efflux pump mechanisms as the primary mechanisms of bacterial resistance to tigecycline [45].

### 3.3. PCR for tet(X) Genes

In this study, PCR assays were developed for the detection of five *tet(X)* variant genes from different samples, namely *tet(X1*), *tet(X2*), *tet(X3*), *tet(X4)*, and *tet(X5)*, which were also used as positive controls for each gene. Premier 5.0 (PRIMIER Biosoft International, Palo Alto, CA, USA) was used to design specific primers for *tet(X1)*, *tet(X2)*, *tet(X3)*, and *tet(X4)*, as well as a universal *tet(X5)* primer set. With a conventional PCR machine and SYBR green-based assay, the accumulation of amplicons in a reaction was monitored over time. Target genes were identified based on certain amplicons’ melting peak form and Tm. Except for *tet(X1)* (65.2%), all other *tet(X) (tet(X1)*, *tet(X2)*, *tet(X3)*, and *tet(X4))* gene compositions are like *tet(X)*, about 85.1% to 99.8%. Though each gene has its pair of primers, the high level of similarity on the *tet(X)* variant does pose a challenge in designing specific primers, especially *tet(X2)* and *tet(X)*, since they are the same except for two nucleotides [46]. Of the 100 CRE clinical isolates studied, none yielded *0 (tet(X1))*, *tet(X2)*, *tet(X3)*, *tet(X4*), and *tet(X5)* using PCR. In this study, *tet(X)* genes were not identified as the primary mechanism of tigecycline resistance; even though tigecycline resistance is notably high from the sample of this study, it is still lower than 15%, which means that there is another resistance mechanism that confers tigecycline resistance such as efflux pumps and mutations in genes like *ramR*, *acrR*, *adeS*, *rrf*, and *rpoB.* These findings align with a study conducted by [17], where real-time PCR was performed to detect *tet(X)* genes in hospital isolates. The justification for the similar results might be that other countries reserve tigecycline drugs, and they are used in exceptional cases, such as South Africa and China. The results of this study are contrary to the study by Zhou et al. [47], where *tet(X)* genes were detected in hospital isolates. The difference is about 7.4%; this could be due to the difference in CLSI guidelines on tigecycline, and *tet(X)* genes were not identified as the primary mechanism of tigecycline resistance. This suggests that there are resistance mechanisms acquired by pathogens that have not been investigated.

### 3.4. Risk Factors Associated with Tigecycline

Few studies have assessed the risk factors associated with tigecycline resistance in CRE among hospital isolates. According to the literature available, tigecycline resistance may be conferred by many factors, including prior exposure to antibiotics, as the use of different drugs (particularly tetracycline) can confer tigecycline resistance in patients, and being in an ICU, where you might be exposed to a variety of resistant organisms. Multivariable analysis indicated that nasal catheter use and exposure to antibiotics such as penicillin and fluoroquinolones were independent predictors for acquiring TCREC [47]. This study aimed to determine the risk factors associated with tigecycline resistance in CRE using patient history and demographic characteristics. The correlation between tigecycline resistance and wards was weak, indicated by r = 0.4182, and the *p*-value of 0.1631 indicated that the association was not statistically significant. This suggests that being admitted to clinical wards does not necessarily mean that patients would be resistant to tigecycline, even though patients admitted to the ICU (25.1%) showed an increased resistance than those admitted to other wards. Most patients admitted to the ICU are more vulnerable to MDR pathogens, which confer resistance to tigecycline. Another study reported that patients with urinary catheterization admitted to the intensive care unit (ICU) had an increase in tigecycline-resistant infections. For age, a statistically significant *p*-value of 0.001 suggests a strong relationship between tigecycline resistance and age groups. The r = value of 0.9501 indicates a strong correlation. There was a notable tigecycline resistance in older patients compared to neonates—as people grow, they tend to use different antibiotics, and most older patients are immunocompromised and are vulnerable to many pathogens with resistant strains. This suggests that age is a critical factor in tigecycline resistance. One of the most prevalent tigecycline risk factors is the demographic age of the patient; elderly patients are at a higher risk of acquiring tigecycline resistance due to their weakened immune systems and a higher likelihood of various comorbidities [48,49]. There was a positive correlation between tigecycline resistance and pre-exposure to antibiotics, as indicated by a statistically significant *p*-value of 0.0478. Undergoing specific invasive procedures also might contribute to tigecycline resistance, as indicated by a statistically significant *p*-value of 0.0478. Patients having central venous catheterization, mechanical ventilation, or urine catheterization are more vulnerable. These procedures may bring pathogens into sterile conditions, facilitating the acquisition of MDR organisms [40]. A non-significant *p*-value of 0.3679 was found between the length of hospitalization and tigecycline resistance. This suggests that hospitalization duration might not be a direct risk factor for tigecycline resistance, even though patients with prolonged, unnecessary stays might acquire tigecycline resistance more often than patients with standardized hospitalization [50,51]. Jiang et al. [29] discovered that using nasal catheters and exposure to medications such as penicillin and fluoroquinolones were independent predictors of tigecycline resistance. The prevalence of tigecycline resistance in this study was 7.1%, higher than Jiang et al.’s findings in China, where they evaluated tigecycline resistance in CRE. It is recommended that tigecycline resistance be reduced by practicing reasonable antibiotic use and minimizing invasive procedures [46]. Socioeconomic factors also play a crucial role in infection rates and resistance; poor living conditions are associated with high tigecycline resistance in CRE isolates. This is likely due to age-related changes in pharmacokinetics and pharmacodynamics and the higher prevalence of underlying conditions in the elderly population. Healthcare professionals should exercise vigilance when prescribing tigecycline to older patients and closely monitor adverse events like thrombocytopenia, particularly among those with three or more risk factors.

## 4. Materials and Methods

### 4.1. Study Area

Nelson Mandela Academic Hospital is a large provincial government-funded hospital in central Mthatha in South Africa in the Eastern Cape. It is a tertiary teaching hospital and part of the Mthatha Hospital Complex. The study was conducted in this hospital and surrounding hospitals such as NMAH Mthatha Regional Hospital, Zithulele Hospital, St. Elizabeth Hospital, Malizo Phehle Hospital, Madzikane Hospital, etc.

### 4.2. Study Design

The present study was a retrospective study focusing on CRE bacterial isolates. The aim was to conduct a molecular analysis of tigecycline resistance in CRE in hospital isolates from Mthatha Hospital and surrounding hospitals. This is a sub-study of the study “Epidemiology, risk factors and molecular analysis of carbapenem-resistant *Enterobacteriaceae* (CRE) in Mthatha, Eastern Cape, South Africa”, which was conducted by Vasaikar et al. [52]. No direct intervention or interaction with any participant occurred as previously non-identified records of CRE data were used. From the previous study, written informed consent was obtained for all adult cases and controls, and parental consent was obtained for child cases and controls. Participants were interviewed by a medical practitioner and research assistant. A clinical research form (CRF) questionnaire was completed upon obtaining a patient’s consent. Factors associated with a clinical sample positive for CRE and CSE in participants who received medical care in NMAH and MRH were studied using a case-control study. Participants prospectively identified by the National Health Laboratory Service (NHLS) microbiological laboratory (Mthatha branch) of each participating hospital with a clinical sample yielding CRE or CSE were eligible for the study and were selected from the laboratory register of the same hospital.

### 4.3. Study Setting

The main study was carried out from April 2019 to December 2024 in South Africa in the Eastern Cape province, Mthatha region, using bacterial isolates of participants who received medical care at NMAH and MRH during the study period. The data used were retrospective and sought from NHLS in Mthatha.

### 4.4. Study Population and Sampling Method

The population criteria for the study were all hospital or clinical tigecycline-resistant isolates (determined using the Vitek2^®^ system (bioMérieux, Marcy l’Etoile, France)) obtained from Mthatha NHLS during the study period from April 2019 to October 2024.

Inclusion criteria: Carbapenem-resistant *Enterobacterales* (CRE) were defined or confirmed when the organism identified showed resistance to one or more carbapenem antibiotics using the Vitek2^®^ system (bioMérieux, Marcy l’Etoile, France). The confirmation of CRE was performed with the Epsilometer test (E test) (bioMérieux, Marcy l’Etoile, France). CRE isolates for this study were selected based on whether they showed tigecycline phenotypic resistance. Sample size: The study is a retrospective study of genes responsible for carbapenem-resistant *Enterobacterales* (CRE) in Mthatha Hospital and correlating predisposing factors, conducted by Prof. Vasaikar.

Exclusion criteria: All the CRE isolates were without tigecycline phenotypic resistance and non-CRE isolates like *Acinetobacter baumannii*.

### 4.5. Data Treatment and Analysis

All variables from the questionnaires and laboratory investigation were captured and coded in Microsoft Excel and exported to SPSS version 20 for analysis. The validity and reliability of the questionnaire data were determined using SPSS version 20. Numerical variables were explored using the Shapiro–Wilk test, histograms, and box-and-whisker plots. The median and interquartile range (IQR) were used to summarize data that were not normally distributed. The chi-squared test and Fisher’s exact test were used to compare the association of two categorical variables depending on the value of expected frequencies, where if 20% or more of the cells have expected frequencies of <5 or any cell has expected frequencies of “0”, Fisher’s exact test was used. Descriptive statistics and logistical regressions were used to estimate the crude ratios with a 95% confidence interval (CI) for the variables. The chi-square test was used to test the hypothesis since the relationship between two or more variables was investigated; the *p*-value was greater than 0.05 (0.98), which means that there was no statistical significance, so the null hypothesis was rejected and the alternative hypothesis was accepted. The significance level was 5%; a *p*-value of less or equal to 0.05 was considered significant.

### 4.6. Ethical Considerations

An ethical clearance certificate was obtained from the Faculty of Medicine and Health Science Ethics Committee with ethical approval number 026/2024. Hospital approval was obtained through the Department of Health. No consent was required from participants as this was a retrospective study. Ethical consideration followed the non-identification principle. Identifiers’ names and surnames (personal information including the patient’s identity number) were removed, and files were created so that any researcher could access them without violating confidentiality. Study IDs were made and used instead. Collected data were stored in a Microsoft Office spreadsheet, which was stored in a password-protected folder on a password-protected laptop. The only individuals with access to this were the researcher and research supervisor. This research aligns with research objective number 3 of a study conducted by Prof. Vasaikar, who was the principal investigator of the research. Ethics certificate 080/2017 and the letter extension were issued on 21 July 2023. Prof. Vasaikar obtained and provided the gatekeeper with a permission letter.

### 4.7. Methodological Design

#### 4.7.1. Antibiotic Susceptibility Testing (E-Test)

The susceptibility test on 100 CRE isolates was performed using the Vitek2^®^ system (bioMérieux, Marcy l’Etoile, France), and phenotypic resistance was confirmed using an Epsilometer (E-test) (bioMérieux, Marcy l’Etoile, France) according to the manufacturer’s instructions and following Clinical and Laboratory Standards Institute (CLSI) [53].

#### 4.7.2. DNA Extraction

About three to four similar-looking colonies grown overnight from MacConkey agar were suspended in 30 mL sized vials containing saline. Then, 10 mL of ZYMO Quick-DNA^TM^ Bacterial Miniprep Kit bacteria lysis butter was added to the vials and vortexed briefly for 10 s to mix thoroughly. They were heated at 95 degrees for 10 min in a heat block to burst open and lyse the cells and centrifuged for about 10 min at 13,000× *g* to obtain the pellet. The supernatant was pipetted into sample tubes and loaded into the ZYMO Quick-DNA^TM^ nucleic acid isolation kit following the manufacturer’s instructions (Zymo Research, Irvine, CA, USA).

#### 4.7.3. *tet(X)* Gene Detection Using Conventional PCR

PCR assays were developed for the detection of five *tet(X)* variant genes from different samples, namely *tet(X1), tet(X2), tet(X3), tet(X4),* and *tet(X5)*, and *E. coli* ATCC 25922 tigecycline-resistant strain was used as a positive control. Primer Premier 5.0 (PRIMIER Biosoft International, Palo Alto, CA, USA) was used to design specific primers for *tet(X1)*, *tet(X2)*, *tet(X3)*, and *tet(X4)*, as well as a universal *tet(X5)* primer set (Table 6).

Primer BLAST 2.14.0 software was used to verify and check primer specificity (using the website https://www.ncbi.nlm.nih.gov/tools/primer-blast/, accessed on 12 March 2024). Conventional simplex PCR analysis and agarose gel electrophoresis were used to verify the primer design with the following conditions. The summary of PCR conditions used are summarized in Table 7.

PCR assays were conducted in 20 µL reaction volumes containing 10 µL of 2 PowerUp master mix (Thermo Fisher Scientific, Waltham, MA, USA), 0.8 µL of each primer (10 mol/L), 6.4 µL of nuclease-free water, and 2 µL of DNA template. Three technical replicates were conducted for each sample. The gel electrophorese concentration was 1.5%, the agarose table was 1.5 g, and the buffer was 100 mL with 4 µL DNA and 6 µL of loading dye mixed with red gel bands. DNA bands were observed on a UV Transilluminator.

## 5. Conclusions

CRE gradually evolves, posing a significant threat to patients of all ages. Thus, early detection of carbapenemase production in clinical infections, carriage states, or both is essential to prevent hospital-based outbreaks. More research is needed to explore the specific factors contributing to the rise in CRE infections, the effectiveness of current control measures, and the development of new strategies to prevent and manage these infections. As using tigecycline is the last resort in the treatment of CRE infection, the high rate of tigecycline resistance in this study is alarming for the healthcare system in South Africa, particularly in Eastern Cape Mthatha and surrounding hospitals. Tigecycline combination therapy is recommended when treating critically ill patients, and clinicians should not use broad-spectrum antibiotics hastily, especially in experimental treatments, to minimize tigecycline resistance in CRE. *tet(X)* genes were not identified as the primary mechanism of tigecycline resistance. The risk factors associated with tigecycline resistance in CRE include age, pre-exposure to antibiotics, prolonged hospitalization, and undergoing invasive procedures.

## Figures and Tables

**Table 1:** Prevalence of carbapenem-resistant *Enterobacterales* in Mthatha and surrounding hospitals.

CRE	Percentage (%)
*Klebsiella pneumoniae*	53.4%
*Escherichia coli*	6.7%
*Enterobacter* spp.	20.5%
*Citrobacter* spp.	19.4%

**Table 2:** The distribution of positive carbapenemase gene loci according to bacterial species.

Species	VIM	KPC	NDM	OXA-48	Not Detected
*Citrobacter* spp.	0	0	50%	25%	25%
*Enterobacter* spp.	0	0	45.6%	27.8%	27.%
*Escherichia coli*	0	0	60%	40%	0
*Klebsiella pneumoniae*	0	0	60.2%	29.1%	11.3%

**Table 3:** Species of carbapenem-resistant *Enterobacterales* according to the specimen type in Mthatha Hospital.

		Specimen	Type				
Species	Abscess	Blood Culture	Pus Swab	Sputum	Urine	Tissue	Other
*Citrobacter* spp.	0	0	8.3%	14.3%	5.9%	0	0
*Enterobacter* spp.	0	13%	33.3%	33.3%	41.2%	0	80%
*Escherichia coli*	0	4.35%	50%	14.3%	5.9%	0	0
*Klebsiella pneumoniae*	100%	86.9%	41.7	71.4%	41.2%	100%	20%

**Table 4:** A chi-square table showing tigecycline resistance by age group at Mthatha and surrounding hospitals.

Chi-Square Tests			
	Value	Df.	Asymptotic Significance
Pearson Chi-square	47.085 ^a^	64	0.944
Likelihood Ratio	34.491	64	0.999
N of Valid Cases	70		

^a^ Ninety-eight cells (99.0%) have an expected count of less than 5. The minimum expected count is 0.1.

**Table 5:** Association between tigecycline resistance and risk factors by *p*-values and R-values.

		Tigecycline Susceptibility				
Tigecycline Resistance Risk Factor	No. of CRE (%)	S	R	I	*p*-Value	R-Value
**Ward**					0.1631	0.4182
Accident and Emergency	1.43	100	0	0		
Adult Care	1.43	100	0	0		
Casualty	2.86	100	0	0		
Female Surgical	4.29	100	0	0		
Gyno	1.43	100	0	0		
ICU	5.71	75	25	0		
Male Surgical	14.29	100	0	0		
Maternity	8.57	100	0	0		
Neonatal	24.29	94.11	0	5.88		
Trauma	2.86	100	0	0		
Not stated (unknown)	1.43	100	0	0		
OPD	11.43	100	0	0		
Pediatrics	20	100	0	0		
**Age**					0.0011	0.9501
0–1	42.86	98.57	0	1.43		
2–15	2.8	100	0	0		
16–29	11.42	100	0	0		
30–43	8.57	98.57	1.43	0		
44–56	8.57	100	0	0		
57–70	11.42	100	0	0		
71–83	7.14	100	0	0		
84–96	5.71	100	0	0		
97–109	0	0	0	0		
**Gender**					0.001	0.991
Males	47.88	97.05	2.94	100		
Females	50.7	100	100	2.94		
Not stated	1.43	100	100	100		
**Prior exposure** **to antibiotics**					0.0478	0.967
Antibiotic intake	75.71	98.11	1.88	0		
No antibiotics taken	24.29	94.41	0	5.88		
**Invasive procedure**						
Underwent procedure	5.63	75	25	0	<0.0001	
No procedure	94.37	98.51	1.49	0		
**Hospitalization duration**					0.3679	
Prolonged	10	98.69	1.41	0		
Standardized	90	98.68	0	1.41		

**Table 6:** Primers for PCR detection and PCR amplification for *tet(X)* genes.

Gene	Sequences 5’-3’	Band Size	Annealing Temperature (°C)	References
*tet(X1)*	F-CAGCGTTTCCGAGTTCTTGA R -GGACGATTACTCTTCCAAGGCT	141	80.6	[23]
*tet(X2)*	F -TGCGGCTAATGGCATCTCAC R- GCTGCTACACATGACAACGTCGT	227	81.6	[23]
*tet(X3)*	F- GTGGATGCTTTGCTATTGTCTGA R -TCTGTTGATTCGTCCTGCGTAT	125	79.5	[23]
*tet(X4)*	*tet(X4)*-F TTGGGACGAACGCTACAAAG *tet(X4)*-R CATCAACCCGCTGTTTACGC	93	55	[17]
*tet(X5)*	F- TGCCGTTGACCTACACAAAGG R -TGTCAAAACGATTTTCG GGTC	161	80.9	[23]

**Table 7:** PCR conditions.

Step	Temperature (°C)	Time	Cycles
Activation	50	2 min	1
Denaturation	95	3 min	40
Annealing	95	30 s	1
Elongation	60	30 s	1
Final Elongation	72	30 s	1
Storage	4	Indefinite	n/a

## Data Availability

The data presented in this study are available from the corresponding author upon reasonable request.
